# Degeneration of core neural tracts for emotional regulation in a patient with traumatic brain injury: A case report

**DOI:** 10.1097/MD.0000000000024319

**Published:** 2021-01-29

**Authors:** Eun Bi Choi, Sung Ho Jang

**Affiliations:** Department of Physical Medicine and Rehabilitation, College of Medicine, Yeungnam University, Daegu, Republic of Korea.

**Keywords:** depression, diffusion tensor tractography, disinhibition, neuronal degeneration, traumatic brain injury

## Abstract

**Rationale:**

Several brain structures, including the orbital prefrontal cortex, ventrolateral prefrontal cortex, dorsolateral prefrontal cortex, amygdala, and anterior cingulate cortex, are considered key structures in the neural circuitry underlying emotion regulation. We report on a patient showing behavior changes and degeneration of core neural tracts for emotional regulation following traumatic brain injury (TBI).

**Patient concerns:**

A 51-year-old male patient suffered an in-car accident. The patient lost consciousness for approximately 30 days, and his Glasgow Coma Scale score was 3. He underwent stereotactic drainage for traumatic intraventricular and intracerebral hemorrhages. At approximately 6.5-year after onset, he began to show disinhibition behaviors such as shouting with anger, which worsened over time. At approximately 8-year after onset, he showed severe depression signs and disinhibition, including violence.

**Diagnoses:**

The patient who showed delayed-onset behavioral changes (disinhibition and depression).

**Interventions:**

Diffusion tensor imaging data were acquired at 3 months and 8 years after TBI onset.

**Outcomes:**

The patient showed degeneration of core neural tracts for emotional regulation that was associated with delayed behavioral changes following TBI. On both 3-month and 8-year diffusion tensor tractographies (DTTs), the right dorsolateral prefronto-thalamic tract, ventrolateral prefronto-thalamic tract, orbital prefronto-thalamic tract, uncinate fasciculus, and both cinguli were reconstructed whereas other neural tracts were not reconstructed. Compared with the 3-month DTT, all reconstructed neural tracts on the 8-year DTT were narrow, except for the left cingulum, which showed new transcallosal fibers between both anterior cingula. The fractional anisotropy and tract volume of all reconstructed neural tracts were lower on the 8-year DTT than the 3-month DTT, except for the tract volume of left cingulum.

**Lessons:**

The evaluation of dorsolateral, ventrolateral, and orbital prefronto-thalamic tract, uncinate fasciculus, and cingulum using follow-up DTTs is useful when a patient with TBI shows delayed-onset behavioral problems.

## 1 Introduction

Several brain structures, including the orbital prefrontal cortex (OFC), ventrolateral prefrontal cortex (VFC), dorsolateral prefrontal cortex (DFC), amygdala, and anterior cingulate cortex (ACC), are considered key structures in the neural circuitry underlying emotion regulation.^[1–3]^ The recently developed diffusion tensor tractography (DTT), an imaging approach derived from diffusion tensor imaging (DTI) data, has enabled evaluation of the neural tracts in the circuitry underlying emotion regulation: the dorsolateral prefronto-thalamic tract (DLPTT); DFC-mediodorsal nucleus (MD) of the thalamus, the ventrolateral prefronto-thalamic tract (VLPTT); VFC-MD, the orbital prefronto-thalamic tract (OPTT); OFC-MD, the uncinate fasciculus; amygdala-frontal lobe, and the cingulum.^[4–8]^

Many studies have reported on neural degeneration following traumatic brain injury (TBI).^[9–14]^ Two kinds of neuronal degeneration occurring after primary direct insult to neuronal cells in the central nervous system have been suggested: primary degeneration—death of neurons and glial cells as an early consequence of the primary pathological insult; secondary degeneration—degeneration of neuronal cells caused by noxious factors released from neurons that were damaged by the primary direct insult.^[9,15]^ It is challenging to differentiate secondary degeneration from primary degeneration based on the time after injury because there is no absolute time when the primary damage evolves into delayed effects following the TBI.^[9,15]^ On the other hand, previous studies have reported on delayed-onset neurological manifestations that began far after the onset of TBI.^[9,10,14]^ A few studies have used DTI to report the degeneration of various neural tracts in the brain, such as the spinothalamic tract, fornix, cingulum, and corticoreticulospinal tract.^[9,12–14]^ However, no study on the degeneration of core neural tracts for emotional regulation has been reported.

In this study, by using DTT, we report on a patient who showed degeneration of core neural tracts for emotional regulation that was associated with delayed behavioral changes following TBI.

## 2 Case presentation

**Figure 1:**
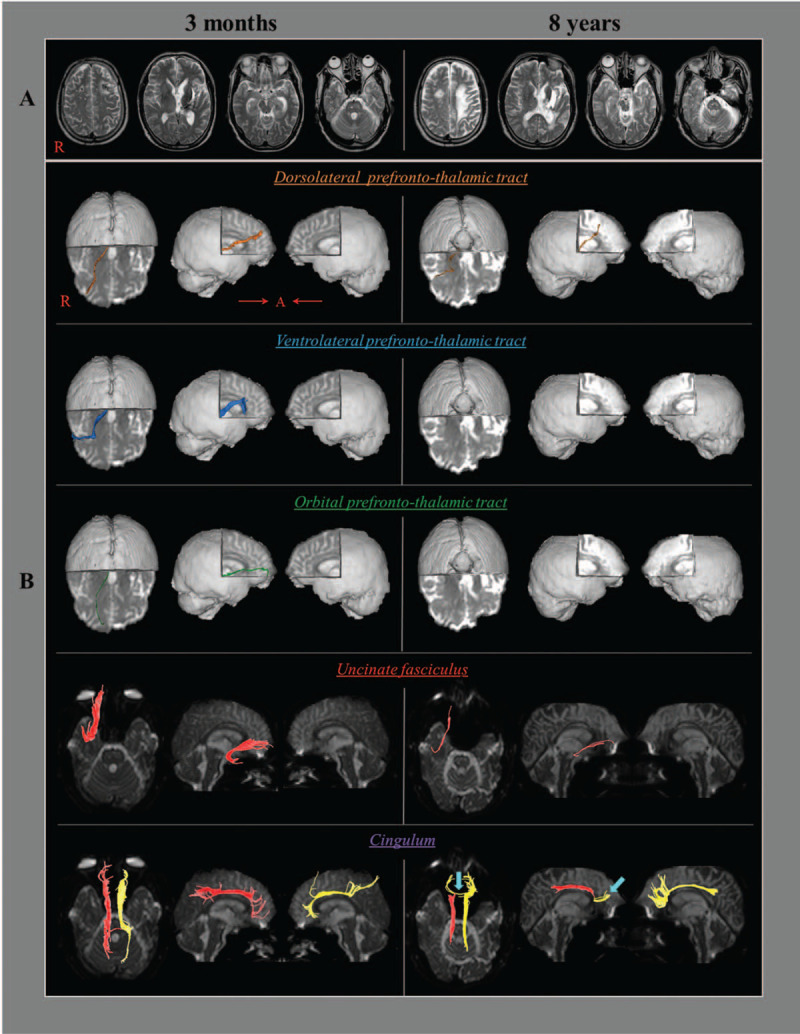
(A) Brain magnetic resonance images obtained at 3 months and 8 years after traumatic brain injury onset show leukomalactic lesions in the left basal ganglia and subcortical white matter. (B) On both 3-month and 8-year diffusion tensor tractographies (DTTs), the right dorsolateral prefronto-thalamic tract (DLPTT), the ventrolateral prefronto-thalamic tract (VLPTT), the orbital prefronto-thalamic tract (OPTT), the uncinate fasciculus, and both cinguli are reconstructed, whereas other neural tracts are not reconstructed. All reconstructed neural tracts on 8-year DTT are narrow compared with the 3-month DTT, except for the left cingulum which shows new transcallosal fibers (arrow) between both anterior cingula.

A 51-year-old male patient suffered an in-car accident: while driving a sedan, his car was struck from behind by another sedan. The patient lost consciousness for approximately 30 days and experienced posttraumatic amnesia for approximately 3 months from the accident. His Glasgow Coma Scale score was 3 when he arrived at the emergency room of a university hospital. He underwent stereotactic drainage for the traumatic intraventricular and intracerebral hemorrhages in the left basal ganglia (Fig. 1A) and rehabilitation for 2 months, beginning 2 months after onset, at the rehabilitation department of the same university hospital. However, he did not show behavioral disinhibition. At approximately 6.5 years after onset, he began to show disinhibition behaviors, such as shouting with anger, which worsened over time. At approximately 8 years after onset, he exhibited severe disinhibition, including violence as follows: he sometimes confronted his caregiver with swearing and offensive language over trivial matters, he shouted in response to received questions, and he removed his shirt frequently. He also showed severe depressive symptoms such as crying when remembering his past and, on testing, exhibited severe depression (Beck Depression Inventory-II score: 41 [full score: 63]).^[16]^ The patient's sister provided signed, informed consent for his participation in the study, and institutional review board approved the study protocol.

DTI data were acquired at 3 months and 8 years after TBI onset using a 6-channel head coil on a 1.5T Philips Gyroscan Intera magnetic resonance imaging scanner (Philips, Ltd., Best, the Netherlands) with 32 non-collinear diffusion sensitizing gradients by performing single-shot, spin-echo planar imaging. Imaging parameters were as follows: acquisition matrix = 96 × 96; reconstructed to matrix = 192 × 192; field of view = 240 mm × 240 mm; repetition time = 10,398 ms; echo time = 72 ms; echo-planar imaging factor = 59; *b* = 1000 s/mm^2^; number of excitations = 1; slice thickness = 2.5 mm. Eddy current and image distortion corrections and fiber tracking were performed using the Oxford Centre for Functional Magnetic Resonance Imaging of the Brain (FMRIB) Software Library (FSL; https://fsl.fmrib.ox.ac.uk/fsl). For analysis of the prefronto-thalamic tracts,^[4]^ the seed region of interest (ROI) was placed on the known anatomical location of the mean diffusivity on the coronal image and each target ROI was delineated as follows: OFC as Brodmann areas (BAs) 11, and 13 on the axial image; VFC as BAs 44, 45, and 47 on the coronal image; and DFC as BAs 8, 9, and 46 on the coronal image. For the reconstruction of the uncinate fasciculus and cingulum, DTI-Studio software (CMRM, Johns Hopkins Medical Institute, Baltimore, MD) was used. Regarding the evaluation of the uncinate fasciculus, the seed ROI was drawn in the frontal part of the uncinate fasciculus on the coronal image through the genu of the corpus callosum and just anterior to the anterior horn of the lateral ventricle. The target ROI was set on the white matter at the most anterior part of the temporal stem on the coronal image.^[17]^ For analysis of the cingulum, the seed ROI was placed on the middle portion of the cingulum, and the target ROI was placed on the posterior portion of the cingulum on the coronal plane.^[6]^ Fiber tracking was performed using a fractional anisotropy (FA) threshold of >0.2 and a direction threshold of <60°.

On both 3-month and 8-year DTTs, the right DLPTT, VLPPT, OPTT, uncinate fasciculus, and both cinguli were reconstructed, whereas other neural tracts were not reconstructed (Fig. 1B). Compared with the 3-month DTT results, all reconstructed neural tracts on 8-year DTT appeared narrow except for the left cingulum, which showed new transcallosal fibers between both anterior cingula. The FA values of all reconstructed neural tracts were lower on the 8-year DTT than on the 3-month DTT. In addition, the tract volume (TV) values of all reconstructed neural tracts, except for the left cingulum, were lower on the 8-year DTT than on the 3-month DTT (Table 1).

**Table 1:** Diffusion tensor tractography parameters of the prefronto-thalamic tract, uncinate fasciculus, and cingulum in a patient with traumatic brain injury.

	3 months post-onset				8 years post-onset			
	FA		TV		FA		TV	
	Rt	Lt	Rt	Lt	Rt	Lt	Rt	Lt
Prefronto-thalamic tract								
DLPTT	0.35	–	257	–	0.30	–	137	–
VLPTT	0.35	–	687	–	–	–	–	–
OPTT	0.40	–	165	–	–	–	–	–
Uncinate fasciculus	0.44	–	980	–	0.42	–	165	–
Cingulum	0.46	0.50	1293	1038	0.42	0.47	484	1269

DLPTT = dorsolateral prefronto-thalamic tract; FA = fractional anisotropy; Lt = left; OPTT = orbital prefronto-thalamic tract; Rt = right; TV = tract volume; VLPTT = ventrolateral prefronto-thalamic tract.

## 3 Discussion

In this study, we performed follow-up DTT of the neural tracts for emotional regulation in a patient who showed delayed-onset behavioral changes (disinhibition and depression), which started approximately 6 years after the patient's head trauma. The FA values of all reconstructed neural tracts were lower on the 8-year DTT than on the 3-month DTT. The TV values of all reconstructed neural tracts, except the left cingulum, were also lower on the 8-year DTT than on the 3-month DTT. These changes coincided with the reconstruction configuration changes observed between the 3-month and 8-year DTTs. Although the TV value of the left cingulum was mildly higher, this appeared to be the effect of an increase in transcallosal fibers, which might be indicative of a compensatory phenomenon for discontinuation of the left anterior cingulum and suggestive of cholinergic reinnervation.^[18]^ The FA parameter represents the state of white matter organization by indicating the degree of directionality and integrity of white matter microstructures. By contrast, the TV indicates the number of voxels included in a neural tract, which is deemed representative of the number of fibers within that tract. Decreases in FA and TV values indicate a decrease in the directionality and the number of neural fibers of a neural tract, respectively.^[19,20]^ In this study, the above changes in the neural tracts for emotional regulation appear to indicate neural degenerations of these neural tracts during the post-onset period. Considering that behavioral changes in this patient started at 6 years after onset, we think that the changes in the behavior of this patient are related to the degeneration of these neural tracts, although it is not clear when this degeneration may have started.

In conclusion, we demonstrated degeneration of the core neural tracts for emotional regulation in a patient who showed delayed-onset behavioral changes following TBI. Our results suggest that evaluation of these neural tracts using follow-up DTTs is useful when a patient with TBI shows delayed-onset behavioral problems. Since the introduction of DTI, one previous study reported injuries of the neural tracts for emotional regulation in a patient exhibiting severe disinhibition following TBI.^[7]^ The present study is the first to report on delayed degeneration of the neural tracts for emotional regulation following TBI. However, this study is limited because it only involves a single case; thus, the conduct of further complementary studies that include larger numbers of patients is warranted.

## Author contributions

**Conceptualization:** Eun Bi Choi, SungHo Jang.

**Data curation:** Eun Bi Choi.

**Supervision:** SungHo Jang.

**Writing – original draft:** Eun Bi Choi, Sung Ho Jang.

**Writing – review & editing:** Eun Bi Choi, Sung Ho Jang.

## Abbreviations

Abbreviations: ACC = anterior cingulate cortex, BAs = Brodmann areas, DFC = dorsolateral prefrontal cortex, DLPTT = dorsolateral prefronto-thalamic tract, DTI = diffusion tensor imaging, DTT = diffusion tensor tractography, FA = fractional anisotropy, MD = mediodorsal nucleus, OFC = orbital prefrontal cortex, OPTT = the orbital prefronto-thalamic tract, ROI = region of interest, TBI = traumatic brain injury, TV = tract volume, VFC = ventrolateral prefrontal cortex, VLPTT = ventrolateral prefronto-thalamic tract.
